# Molecular Structure and Biosynthesis of Pyoverdines Produced by *Pseudomonas fulva*

**DOI:** 10.3390/microorganisms13061409

**Published:** 2025-06-17

**Authors:** Eri Ochiai, Takeru Kawabe, Masafumi Shionyu, Makoto Hasegawa

**Affiliations:** Graduate School of Bioscience, Nagahama Institute of Bio-Science and Technology, 1266 Tamura-cho, Nagahama 526-0829, Japan; b113048@m.nagahama-i-bio.ac.jp (E.O.); b118041@m.nagahama-i-bio.ac.jp (T.K.); m_shionyu@nagahama-i-bio.ac.jp (M.S.)

**Keywords:** pyoverdine, PVD, *Pseudomonas fulva*, siderophore, non-ribosomal peptide synthetase, NRPS, PvdL, chromophore side chain, microbial iron acquisition

## Abstract

This study explored the biosynthetic mechanisms and structural diversity of pyoverdines (PVDs) produced by *Pseudomonas fulva*. Genomic analysis using antiSMASH identified the PVD biosynthetic gene cluster, although the C-terminal peptide sequence could not be predicted. Subsequent liquid chromatography tandem mass spectrometry (LC-MS/MS) analysis revealed the full peptide structure, including modified residues, such as N-acetylhydroxyornithine and cyclohydroxyornithine, and confirmed the presence of several PVD isoforms with different chromophore side chains. Comparative LC-MS analysis across *Pseudomonas* species demonstrated that *P. fulva* produces unique PVD molecular mass patterns. The bioinformatic and structural modeling of non-ribosomal peptide synthetase PvdL open reading frame 3 revealed that the A2 and A3 adenylation domains are lysine selective. Although their sequences differ from known lysine-specific signatures, AlphaFold3-based structural prediction revealed conserved substrate-binding configurations, suggesting that similar substrate-binding features may have arisen independently. Notably, Thr^297^, a unique residue in the non-ribosomal code, likely plays a key role in lysine recognition. The high degree of sequence similarity between the A2 and A3 domains may reflect domain duplication and could be involved in the diversification of the PVD structure. Further functional and ecological studies are required to assess the physiological significance of *P. fulva* PVDs in microbial iron acquisition.

## 1. Introduction

Iron is an essential element for the survival and proliferation of living organisms. In response to iron limitation, many microorganisms have evolved efficient iron acquisition mechanisms, one of which is the synthesis and secretion of siderophores [1,2,3]. Siderophores chelate iron and transport it into microbial cells, enabling survival in iron-deficient environments. Microorganism-produced siderophores, such as enterobactin from *Escherichia coli* (with a molecular weight (MW) of ~670 Da), desferrioxamine B from *Streptomyces* spp. (MW ~560 Da), and ferrichrome from *Aspergillus* spp. (MW ~740 Da), exhibit a wide molecular weight range from 150 to 2000 Da and display significant structural diversity, including catecholates, hydroxamates, and carboxylates.

Among siderophores, pyoverdines (PVDs), produced by fluorescent *Pseudomonas* species, possess a peptide backbone with sequence diversification that confers a competitive advantage in interspecies survival [4,5,6,7]. The genus *Pseudomonas* includes various fluorescent species, including *P. aeruginosa* [5,8] and *P. syringae* [9], which are pathogenic to animals and plants, respectively. PVDs are key siderophores involved in the establishment and pathogenicity of *P. aeruginosa*, contributing not only to iron acquisition but also to virulence through biofilm formation and phenazine production [8]. In addition, there are soil bacteria, such as *P. fluorescens* [10], *P. putida* [11,12], and *P. protegens* [13], known for their plant-protective properties [14]. In environments with relatively high iron concentrations, fluorescent *Pseudomonas* species produce a low-MW siderophore called pyochelin [1,7]. By contrast, in environments lacking iron, they produce PVDs, characterized by a high iron-binding capacity and efficient receptor-mediated uptake. In soil, fluorescent *Pseudomonas* frequently colonize the rhizospheres of plants. Their efficient siderophore-based iron acquisition systems allow these bacteria to compete with iron-absorbing bacteria and fungi that parasitize plants, thereby suppressing their growth [13,15].

PVDs are peptides comprising three distinct structural components: a dihydroxyquinoline-type chromophore responsible for fluorescence, a peptide chain of 6 to 12 amino acids, and a small dicarboxylic-acid-derived moiety (e.g., succinic acid, malic acid, or their monoamides, glutamic acid, or α-ketoglutaric acid) attached to the chromophore [16]. The length and sequence of the peptide chain vary among strains, correlating with the specificities of the transporters responsible for the uptake of iron-bound PVDs. To date, the amino acid sequences of at least 60 different PVDs have been subjected to chemical characterization [6]. These sequences are not directly encoded by genomic DNA; rather, their core structure is biosynthesized by non-ribosomal peptide synthetases (NRPSs) [17]. These large multi-component enzymatic systems play pivotal roles in the synthesis of diverse complex peptides in bacteria and fungi. Biosynthetic pathways involve the participation of multiple synthetases and auxiliary enzymes, which are responsible for the production of non-proteinogenic amino acid precursors [4,5,18]. It is postulated that certain gene products localize in the periplasm and participate in the maturation of PVDs. However, the structural diversity and evolutionary origins of PVDs remain largely unexplored.

The species *Pseudomonas fulva* has been isolated from a variety of clinical samples [19,20] and environmental sources [21,22]. Some reports indicate the potential for this organism to cause disease, while others highlight its utility in environmental remediation [23] and plant growth promotion [22]. Although *P. fulva* is closely related to the well-studied *P. putida*, they have separate lineages [24,25]. Furthermore, *P. parafulva* and *P. cremoricolorata* are considered as closely related yet distinct species [26]. The peptide sequences of PVDs have been shown to be species-specific within the genus *Pseudomonas* and have historically been used as markers for species identification [11]. Therefore, elucidating the peptide sequence of an unknown PVD can provide valuable insights into the phylogenetic affiliation of the producing *Pseudomonas* strain. In this study, to explore the biosynthetic diversity of PVDs, the NRPSs involved in synthesizing the PVD peptide backbone in *P. fulva*, a relatively understudied species, were identified through genomic analyses. The biosynthetic pathways of *P. fulva* PVDs share similarity with those of other *Pseudomonas* species. However, the C-terminal sequence could not predicted from the conventional non-ribosomal code. To elucidate the structures of *P. fulva* PVDs, tandem mass spectrometry (MS/MS) analysis was conducted, revealing the incorporation of a lysine residue. This prompted an evaluation of the non-ribosomal code in predicting the amino acid selectivity of the NRPS adenylation (A)-domain responsible for incorporating this residue.

## 2. Materials and Methods

### 2.1. Bacterial Culture

*P. fulva* NBRC16639 and other *Pseudomonas* strains were obtained from the National Institute of Technology and Evaluation (NITE), Tokyo, Japan. The strains were maintained in 702 medium and cultured in succinate medium (22 mM KH_2_PO_4_, 34 mM K_2_HPO_4_, 0.4 mM MgSO_4_, 7.6 mM (NH_4_)_2_SO_4_, and 56 mM Na_2_C_4_H_4_O_4_; pH 7.0) for two days at room temperature, with shaking to induce PVD production. The end of the cultivation was determined by visually confirming the fluorescence of the medium under UV light.

### 2.2. Genomic Analysis

The genome of the *P. fulva* NBRC 16639 strain was extracted and purified using a DNA Extraction Kit (QIAGEN N.V., Hilden, Germany). Genomic sequencing, utilizing PacBio sequencing technology, was outsourced to Bioengineering Lab Co., Ltd. (Kanagawa, Japan). The quantification of the genomic DNA from *Pseudomonas fulva* NBRC 16639 was conducted using the QuantiFluor dsDNA System and a Quantus Fluorometer (Promega Co., Madison, WI, USA). The quality of the DNA was assessed by means of capillary electrophoresis, using a 5200 Fragment Analyzer System and an Agilent HS Genomic DNA 50 kb Kit (Agilent Technologies, Inc., Santa Clara, CA, USA). The purification of the DNA was accomplished using DNA Clean Beads (MGI Tech Co., Ltd., Shenzhen, China) at a volumetric ratio of 1.8:1. Shearing was performed with a g-TUBE (Covaris LLC, Woburn, MA, USA) to obtain DNA fragments of approximately 10–20 kbp. For the construction of the library, SMRTbell Prep Kit 3.0 and a SMRTbell gDNA Sample Amplification Kit (Pacific Biosciences of California, Inc. (PacBio), Menlo Park, CA, USA) were used in accordance with the manufacturer’s protocols. The quality of the PCR product was verified by means of electrophoresis using the 5200 Fragment Analyzer System and the Agilent HS Genomic DNA 50 kb Kit. The processing of sequencing libraries was undertaken using a Revio Polymerase Kit (PacBio), and the subsequent execution of the sequencing was conducted on a Revio system (PacBio). The obtained genomic data were annotated using Rapid Annotation and Subsystem Technology (RAST) [27] (https://rast.nmpdr.org/ (accessed on 3 September 2024)).

### 2.3. Purification of PVDs

The purification of the PVDs from *P. fulva* NBRC 16639 and other *Pseudomonas* strains was performed as previously described [28]. Solid-phase extraction was carried out using Strata-X (1 mL, 30 mg) polymeric reverse-phase cartridges (Phenomenex, Torrance, CA, USA). Briefly, the cartridge was washed with 1 mL of methanol and equilibrated with 1 mL of water (H_2_O). The 500 µL supernatant was acidified with 5 µL of formic acid and loaded onto the sorbent. The sorbent was washed with 0.6 mL of water, and the PVD was eluted using 0.6 mL of 30% methanol/H_2_O containing 0.1% formic acid.

### 2.4. LC-MS Analysis

Peptide separation was performed using a COSMOSIL _5_C_18_-AR-II column (2.0 ID × 100 mm; Nacalai Tesque, Inc., Kyoto, Japan) attached to a Vanquish F-Type UHPLC System SII (Thermo Fisher Scientific, Inc., Waltham, MA, USA). The peptides were eluted over a 35 min linear gradient from 10% acetonitrile–H_2_O to 45% acetonitrile–H_2_O (containing 0.1% formic acid) at a flow rate of 0.2 mL/min and a column temperature of 40 °C. Mass analysis of the separated peptides was performed using a Q-Exactive Orbitrap mass spectrometer (Thermo Fisher Scientific) connected to a high-performance liquid chromatography (HPLC) system. Ionization was achieved under standard conditions with a Heated-Electrospray Ionization-II source (at a spray voltage of 3.5 kV, a sheath gas flow rate of 40 L/min, an auxiliary gas flow rate of 10 L/min, a capillary temperature of 350 °C, a probe heater temperature of 300 °C, and an S-lens RF level of 60). Precursor ions were acquired using the full MS/dd-MS^2^ mode (at a resolution of 70,000, an ITmax value of 200 ms, an AGC target of 1 × 10^6^, and a scan range from 1000 to 3000 *m*/*z*). For ions with a high signal intensity, MS/MS fragment peaks were measured by applying stepped collision energies (at normalized collision energies of 20, 25, and 30) to obtain fragmentation data (at a resolution of 35,000, an IT_max_ value of 100 ms, an AGC target of 1 × 10^5^, and a scan range from 200 to 2000 *m*/*z*).

### 2.5. Amino Acid Analysis

Approximately 5 µg of purified PVD (isoform A) was hydrolyzed with 5.7 M hydrochloric acid (800 µL) at 110 °C for 24 h. After the solution was dried, the residue was dissolved in 200 µL of 50 mM hydrochloric acid. The amino acid composition of the sample was analyzed using a High-Speed Amino Acid Analyzer (LA8080, Hitachi High-Tech Co., Tokyo, Japan).

### 2.6. Bioinformatic Analysis

The antiSMASH web server [29] was used to annotate secondary metabolite biosynthetic gene clusters (BGCs) in the *P. fulva* NBRC 16639 genome (https://antismash.secondarymetabolites.org (accessed on 6 May 2024)). We then collected non-ribosomal codes of Lys-selective A-domains and amino acid sequence IDs from the lookup table file (https://dl.secondarymetabolites.org/releases/stachelhaus/1.1/signatures.tsv (accessed on 4 October 2024)), which is used by antiSMASH to predict A-domain substrates. Using the collected sequence IDs, we also collected amino acid sequence data from the NCBI database (https://www.ncbi.nlm.nih.gov (accessed on 7 October 2024)) and extracted the amino acid sequences of Lys-selective A-domains using annotated data obtained from locally installed antiSMASH (https://dl.secondarymetabolites.org/releases/7.0.0/antismash-7.0.0.tar.gz (accessed on 7 October 2024)). We aligned the sequences of the Lys-selective A-domains using MAFFT [30] and estimated the maximum likelihood phylogenetic tree using MEGA 11 [31] with default parameters. The WebLogo sequence logos of the non-ribosomal codes of the Lys-selective A-domains were generated using the WebLogo server [32].

To analyze the interactions between the A2 and A3 domains of the *P. fulva* NRPS PvdL open reading frame (ORF) 3 and lysine molecules, we predicted their three-dimensional (3D) structures complexed with lysine, ATP, and Mg^2+^ using AlphaFold3 [33] installed on a local computer. Because the 3D structure of the Lys-selective A-domain in ε-poly-L-lysine synthetase (Pls-A, PDB ID: 7wew) is known to have an adenylation conformation [34], we selected the predicted structure that has the lowest Root-Mean-Square-Deviation value compared to 7wew from the five candidate structures predicted by AlphaFold3. Schematic drawings of the interactions with the lysine molecule were generated using Molecular Operating Environment (MOE) 2024.06 (Chemical Computing Group ULC, Montreal, QC, Canada, H3A 2R7; 2024).

## 3. Results and Discussion

### 3.1. Characteristics of the P. fulva Genome

The long-read sequencing of the *P. fulva* NBRC 16639 strain’s genome, using PacBio technology, generated a single contig, completing the genome’s assembly with a high degree of accuracy (Table 1). The total genome length was determined to be 4,917,956 base pairs, with a GC content of 61.6%, both consistent with those of other *Pseudomonas* species. The previously reported *P. fulva* DSM 17717 strain has a genome size of 4,770,636 base pairs, indicating that the genome of *P. fulva* NBRC 16639 is 147,320 base pairs longer [35]. Genomic annotation using the RAST server [27] predicted 4541 coding sequences, 144 more than predicted for *P. fulva* DSM 17717. These results indicate genomic expansion in *P. fulva* NBRC 16639, possibly reflecting functional diversification.

### 3.2. Identification of Secondary Metabolite BGCs Using antiSMASH

**Figure 1:**
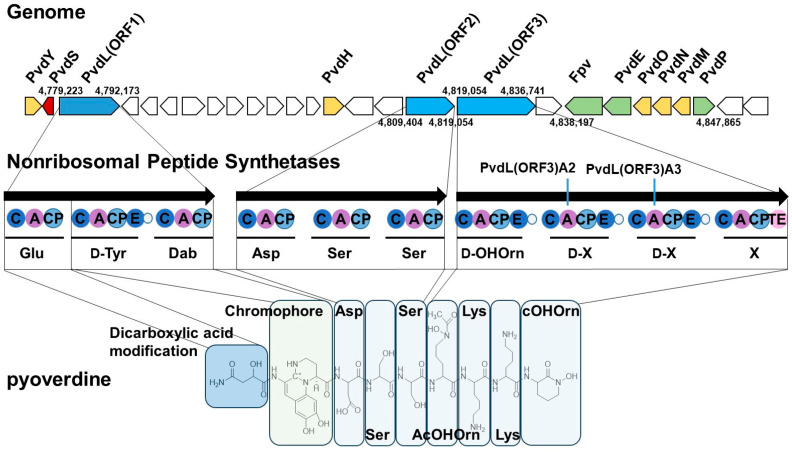
Pyoverdine (PVD) biosynthetic gene cluster (BGC) predicted using antiSMASH analysis. The BGC for the PVD in *P. fulva* NBRC 16639 and the corresponding non-ribosomal peptide synthetase (NRPS) modules are shown. Top: The cluster contains 10 genes involved in PVD biosynthesis, each indicated in a different color on the genomic map. Middle: The NRPS module organization illustrates the predicted functions of individual domains (C, condensation; A, adenylation; CP, peptidyl carrier protein; E, epimerization domain; TE, thioesterase domain) based on antiSMASH predictions [29]. These domains collaboratively activate amino acids and extend the peptide chain in a stepwise manner. Bottom: Structural correspondence between an LC-MS-identified PVD (isoform A) and the predicted peptide sequence, highlighting domain-specific contributions to the final molecular structure.

To analyze the secondary metabolite BGCs in the genome of *P. fulva* NBRC 16639, antiSMASH analysis [29] was performed, and eight BGCs were identified. Among them, the eighth cluster shared a high degree of homology with the PVD BGC of *P. protegens* Pf-5 (Figure 1). This PVD biosynthetic gene cluster is located in the genomic region spanning from 4,777,182 to 4,846,237 base pairs. Ten related genes involved in PVD synthesis were identified within this cluster. These results suggest functional conservation in PVD biosynthesis between *P. fulva* NBRC 16639 and other *Pseudomonas* species known to produce structurally diverse siderophores.

The PvdL enzyme, divided into three ORFs (ORF1, ORF2, and ORF3), is an NRPS responsible for synthesizing the peptide backbone of the PVD, including its chromophore component [5]. Non-ribosomal peptide synthesis involves a modular structure, with each NRPS module activating a specific amino acid and incorporating it into the growing peptide chain [17]. The key domains of NRPSs include the condensation (C)-domain, which catalyzes peptide bond formation; the thiolation (T)-domain, also known as the peptide carrier protein (PCP); and the adenylation (A)-domain, which activates amino acid substrates. Substrate-recognizing residues in the A-domain are collectively referred to as the non-ribosomal code [36,37]. Using these features, antiSMASH [29] can predict the amino acid sequence of synthesized peptides.

The sequences of the synthesized product were predicted as follows: PvdL(ORF1), Glu-D-Tyr-Dab (2,4-diaminobutyric acid); PvdL(ORF2), Asp-Ser-Ser; PvdL(ORF3), D-Orn-D-Xaa1-D-Xaa2-Xaa3. The N-terminal Glu residue is first modified with a myristic or myristoleic acid chain. After cytoplasmic biosynthesis, the product is transported to the periplasm, where the fatty acid chain is removed. The glutamic acid residue is then converted to α-ketoglutaric acid, succinic acid, malic acid or their monoamide, producing several PVD isoforms [18]. PvdH converts L-aspartate β-semialdehyde to Dab [5,18,38], which is condensed with the D-tyrosine top form of the quinoline-type chromophore. Cyclization of the chromophore is thought to occur in the periplasm and involves the enzymes PvdN, PvdO, and PvdP [5,18]. The four N-terminal residues following the chromophore were predicted to be Asp-Ser-Ser-D-Orn (ornithine). However, the identity of the three C-terminal residues, represented by Xaa1-Xaa2-Xaa3, could not be predicted. PvdY acts as an acetyltransferase and catalyzes the acetylation of Nδ-hydroxyornithine in *P. aeruginosa* [39]. This modification forms the iron-binding hydroxamate group. If the peptide chain terminates with an Orn residue, cyclohydroxyornithine (cOHOrn) may be formed, increasing the iron-binding efficiency of the siderophore.

### 3.3. Molecular Structures of the P. fulva PVDs

**Figure 2:**
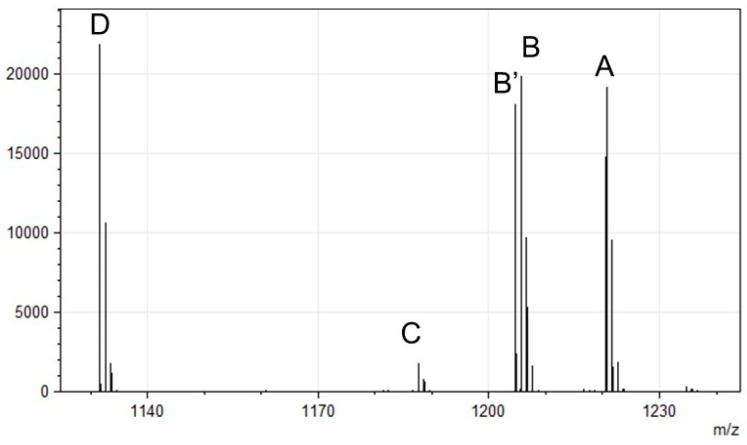
Detection of PVD isoforms using LC-MS. The molecular species corresponding to each peak are referred to as isoforms A, B, B’, C, and D.

To determine the structures of the PVDs produced by *P. fulva*, LC-MS analysis was performed, integrating bioinformatic predictions with analytical chemistry [28,40]. PVD purified from the culture supernatant was analyzed using a Q-Exactive mass spectrometer (Thermo Fisher Scientific, Inc., Waltham, MA, USA), and five molecular ions were detected, corresponding to different PVD isoforms (Figure 2 and Table 2). Each molecular ion was fragmented using MS/MS, producing primarily B ions, Y″ ions (Y ions with two additional hydrogen atoms), and a few A ions [28,40]. These fragments allowed the complete elucidation of the PVD peptide chain structure, confirming the amino acid sequence and modifications.

**Figure 3:**
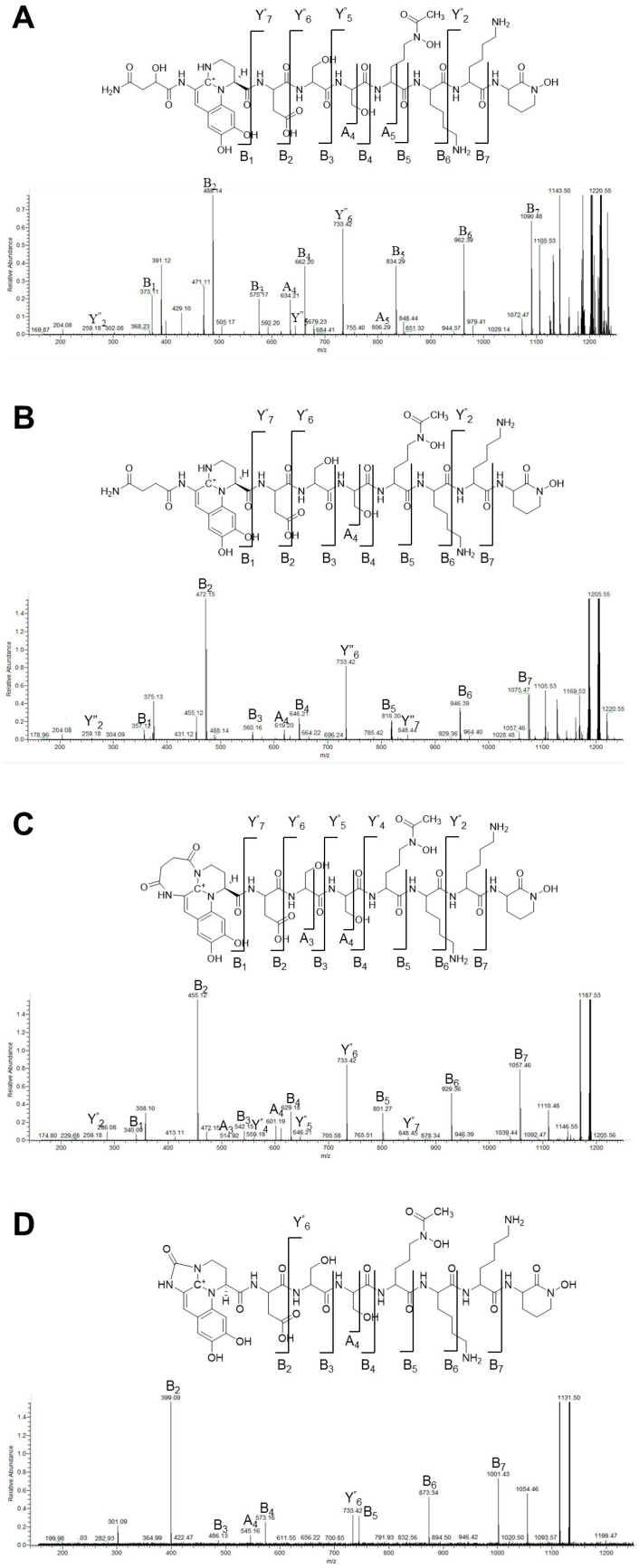
Chemical structures and MS/MS spectra of PVD isoforms A–D. Panels (**A**–**D**) show isoforms A to D, respectively, with their chemical structures (upper panels) and corresponding MS/MS spectra, including fragment ion assignments. Exact mass values of the ions are listed in Appendix A tables. Green lines in the spectra indicate the positions and *m*/*z* values of minor peaks.

MS/MS analysis was performed using the peak for the precursor ion at 1220.56 *m*/*z*, which was assigned to a PVD peptide with a malic acid (Mala) side chain attached to the carboxylic acid group of the hydroxyquinoline chromophore (Figure 3A and Appendix A Table A1). The peptide sequence was assigned as Asp-Ser-Ser-AcOrn(OH)-Lys-Lys-cOrn(OH). Significant B-ion peaks were observed at *m*/*z* 373.1 (B1) and 488 (B2), with an additional peak for the B2 fragment at *m*/*z* 471, corresponding to a dehydrated form due to OH group loss [28]. These peaks confirmed that the first amino acid was Asp. Successive B-ion peaks appeared at *m*/*z* 575, 662, 834, 962, and 1090, completing the peptide sequence. Fragment peaks from C-terminal fragmentation (Y″ ions) were detected at *m*/*z* 259, 733, and 848. These peaks corroborated the sequence deduced from the B-ion series, confirming the accurate assignment of the entire peptide structure. The combined ion fragmentation analysis verified the primary structure of the peptide, including specific modifications, such as acetylated hydroxyornithine (AcOrn(OH)) and cyclohydroxyornithine (cOrn(OH)), essential components of the PVD siderophore.

In the MS/MS fragmentation pattern, characteristic peaks for ions specific to the Mala side chain at *m*/*z* 302 were observed in addition to the peak of the common fragment at *m*/*z* 204, confirming the characteristic fragmentation of the PVD chromophore [16]. These ions are indicative of the hydroxyquinoline-type chromophore attached to Mala, confirming its structural integrity. In addition, the B_1_-ion peak at *m*/*z* 488 yielded a fragment peak at *m*/*z* 429, suggesting the cleavage of the chromophore region rather than the peptide backbone itself [16].

The MS/MS analysis of other detected molecular ion peaks revealed common amino acid sequences but distinct modifications in the side chains of the chromophore (Figure 3B–D and Appendix A Table A2, Table A3 and Table A4), suggesting structural isoforms of PVD: The peak at 1205.54 *m*/*z* was identified as the succinamide-type isoform; the peak at 1204.57 *m*/*z* was assigned to the succinylated-type isoform. The peak at 1187.52 *m*/*z* corresponded to the cyclic succinamide type formed by intramolecular cyclization; the peak at 1131.50 *m*/*z* was designated as an azotobactin-like structure with a five-membered ring added to the chromophore. Both azotobactin and PVDs are known to form stable complexes with metal cations [12,41,42].

### 3.4. Amino Acid Compositions of the P. fulva PVDs

The amino acid composition of *P. fulva* PVD isoform A (*m*/*z* 1220.56) was analyzed (Table 3). Comparative analysis with a standard sample of L-Orn validated this identification. The quantified amino acid composition was Asp, Ser, Orn, and Lys in a ratio of 1.0:2.0:1.12:2.13. Compared with the expected theoretical ratio of 1:2:2:2, based on the identified sequence, the detected ratio of the Orn was lower. This discrepancy likely reflects incomplete recovery due to structural modifications, specifically, acetylated hydroxyornithine (AcOHOrn) and cyclohydroxyornithine (cOrnOH), which may have undergone partial hydrolysis during the analysis, reducing the measured Orn content.

### 3.5. Molecular Mass Distribution of PVDs in Pseudomonas Strains

The molecular masses of the secondary metabolites corresponding to the PVDs in the culture supernatant of Pseudomonas NBRC strains were compared under iron-deficient conditions (Table 4). Two *P. fulva* NBRC strains, 16637 and 16639, exhibited similar molecular mass patterns. However, additional signals were detected at *m*/*z* 1115.47 (azotobactin type -16) for strain 16637 and at *m*/*z* 1238.56 (malic acid amide type +18) for strain 16637, suggesting potential structural variants. These PVDs likely share the same amino acid sequence as that previously identified in strain 16639. An environmental isolate of *P. fulva* displayed a molecular mass pattern identical to that of NBRC 16639. Similarly, *P. parafulva* strains NBRC 16635 and 16636 showed molecular mass patterns that differed from that of *P. fulva* by only two or three mass units, indicating that these closely related species likely produce structurally similar PVDs.

By contrast, one *P. cremoricolorata* strain and nine *P. putida* NBRC strains yielded significantly different molecular mass patterns. Most of the molecular masses detected were consistent with the predicted masses of previously reported PVD sequences. These results suggest that while *P. fulva* appears to produce a limited variety of PVDs with conserved sequences across strains, *P. putida* exhibits greater molecular diversity, reflecting different evolutionary pathways for PVD biosynthesis in these Pseudomonas species.

### 3.6. Bioinformatic Analysis of the Lysine-Incorporating Adenylation Domain of P. fulva PVDs

**Figure 4:**
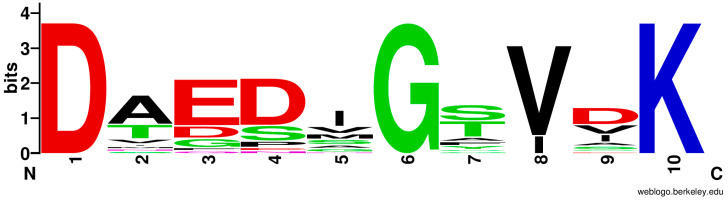
Sequence logo of the non-ribosomal code in Lys-selective A-domains. The residue conservation at each of the 10 canonical positions is shown. Variability is observed at positions 5, 7, and 9, while positions 1, 6, and 10 are highly conserved.

Although the predicted peptide sequence of the product synthesized from PvdL(ORF3) was D-Orn-D-Xaa1-D-Xaa2-Xaa3, MS analysis suggested that both the first and second unknown amino acids (Xaa1 and Xaa2, respectively) were lysine residues. This indicates that the second and third A-domains (A2 and A3) of PvdL(ORF3) must recognize lysine residues. To validate the non-ribosomal code [36,37] of the A2 and A3 domains in *P. fulva* PvdL(ORF3), we compared them with other known Lys-selective A-domains (Table 5). The A2 and A3 domains of PvdL(ORF3) in *P. fulva* share highly similar sequences (95% sequence identity). In particular, the amino acid sequences of the N-terminal core domains of the A2 and A3 domains, both 408 residues long, are almost identical (99% sequence identity). The non-ribosomal code of these A-domains was identified as (1)Asp^191^-(2)Ala^192^-(3)Glu^195^-(4)Asp^233^-(5)His^261^-(6)Gly^263^-(7)Thr^288^-(8)Val^296^-(9)Thr^297^-(10)Lys^486^. The divergence of the non-ribosomal codes among the known Lys-selective A-domains is visualized as a sequence logo in Figure 4, which summarizes the conserved amino acid sequences at each of the 10 positions in the non-ribosomal code. Notably, positions 1, 6, and 10 are highly conserved, corresponding to Asp, Gly, and Lys, respectively. Meanwhile, positions 5 and 9 are variable sites in the known Lys-selective A-domains, and His and Thr have not yet been observed at these sites. The apparent inconsistencies at positions 5 and 9 in the non-ribosomal code of *P. fulva* may explain the failure of antiSMASH to identify lysine as the predicted substrate for the A2 and A3 domains in PvdL(ORF3).

**Figure 5:**
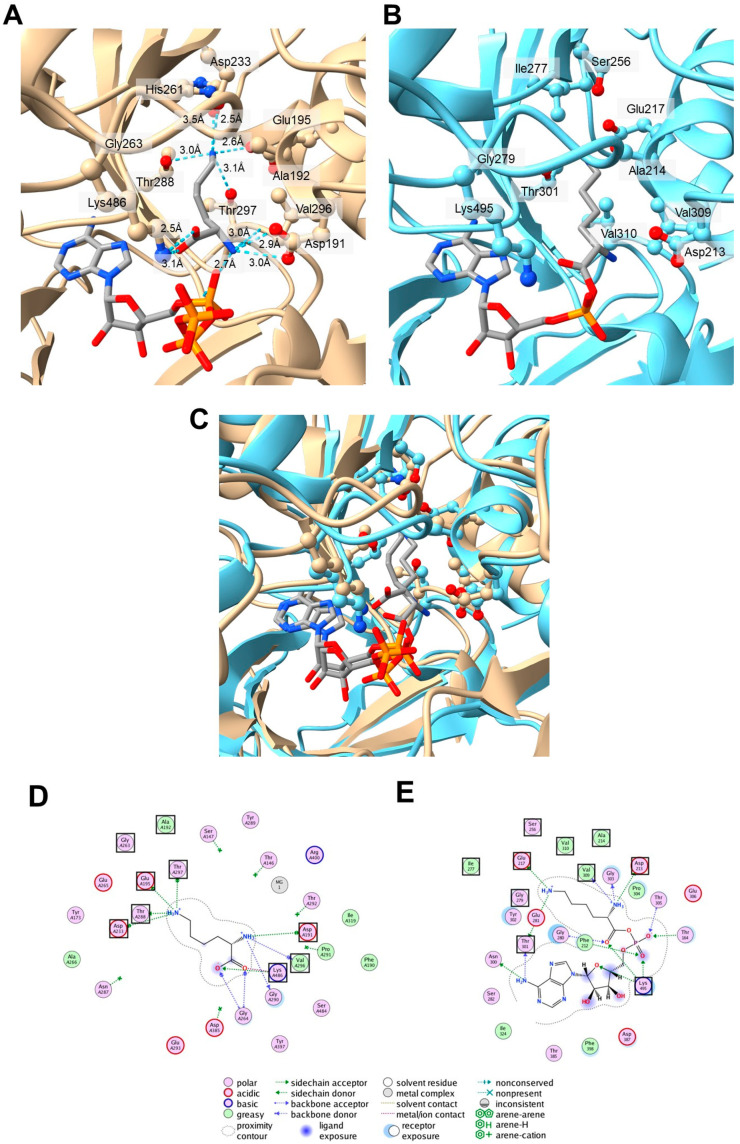
Comparison of the predicted structure of the *P. fulva* PvdL(ORF3) A2 domain, using AlphaFold3, with the X-ray crystal structure of the lysine-selective A-domain of ε-poly-L-lysine synthetase (Pls-A). (**A**) Predicted structure of the A2 domain of Pvd(ORF3) in *P. fulva* (brown). (**B**) Crystal structure of Pls-A (cyan). (**C**) Superimposition of the structures in (**A**,**B**). Non-ribosomal-encoded amino acid residues are shown as ball-and-stick models. Ligand molecules are shown as stick models colored based on the element type. (**D**) Schematic diagram of the interactions between the lysine substrate molecule and the A2 domain of *P. fulva* Pvd(ORF3). To clearly illustrate the mode of the interaction with the lysine substrate molecule, the ATP molecule and Mg^2+^ in the predicted structure of the A2 domain were omitted from the diagram. (**E**) Schematic diagram of interactions between lysyl-adenylate and Pls-A. The amino acid residues that make up the non-ribosomal code are enclosed in black boxes.

**Figure A1:**
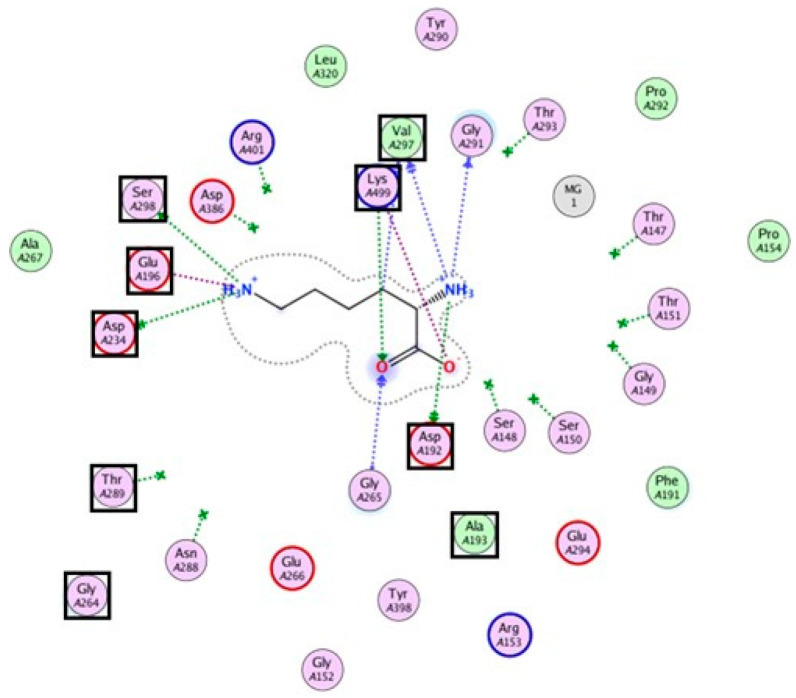
Schematic diagram of the interactions between the lysine substrate molecule and the A3 domain of *P. protegens* Pf-5 PvdD.

Next, the three-dimensional structures of the A2 and A3 domains of *P. fulva* PvdL(ORF3) were predicted using AlphaFold3 [33], and the substrate interaction mode was compared with that of the Lys-selective A-domain in ε-poly-L-lysine synthetase (Pls-A), which structure has been determined using X-ray crystallography [34]. Although the sequence similarity degree between the A2 domains of PvdL(ORF3) and Pls-A is relatively low (35% sequence identity), the predicted structure of the A2 domain in the PvdL(ORF3) complex with lysine and ATP molecules (Figure 5A) showed good agreement with the relative positions of the amino acid residues comprising the non-ribosomal code in the crystal structure of the Pls-A complex with lysyl-adenylate (Figure 5B,C). In the predicted structures of A2 and A3 in PvdL(ORF3), the α-amino group of the substrate forms hydrogen bonds with the side chain of (1)Asp^191^ and the main-chain carbonyl oxygens of (8)Val^296^ and Gly^290^ (Figure 5D). These residues correspond to (1)Asp^213^, (8)Val^309^, and Gly^303^, respectively, in Pls-A (Figure 5E). The carboxyl group of the substrate was stabilized by a salt bridge with (10)Lys^486^, located in the C-terminal subdomain, which is conserved in nearly all NRPS A-domains and plays crucial roles in ensuring precise substrate binding and proper alignment at the reaction site [36]. Furthermore, the side chain of the lysine molecule interacts with (3)Glu^195^, (4)Asp^233^, (7)Thr^288^, and (9)Thr^297^. Of these, (9)Thr^297^ is a unique amino acid type of the non-ribosomal code for A2 and A3 in PvdL(ORF3), as described above. The hydroxyl group of the Thr side chain forms a hydrogen bond with the α-amino group in the side chain of the lysine molecule. This indicates that the unique (9)Thr^297^ of A2 and A3 in PvdL(ORF3) may be important for lysine recognition by A2 and A3 in PvdL(ORF3). The (5)His^261^ residue, which is also a unique amino acid type and not prevalent in the known non-ribosomal code in the Lys-selective A-domains, does not bind to lysine or ATP molecules because (5)His^261^ is buried in the interior of the domain core. A similar outcome was obtained using the predicted structure of the Lys-selective A-domain in PvdD of *P. protegens* Pf-5 (AAY93354), the phylogenetically closest Lys-selective A-domain to A2 and A3 in *P. fulva* PvdL(ORF3). The (5)Asn^262^ residue of the Lys-selective A-domain in the PvdD of *P. protegens* Pf-5 is also not involved in lysine interactions (Appendix B Figure A1). This observation may explain the low sequence conservation degree at position 5 in the non-ribosomal code among Lys-selective A-domains. This trend is further illustrated in the WebLogo plot (Figure 4), which graphically represents the sequence conservation across 10 key residues of Lys-selective A-domains. In this figure, position 5 exhibits significant variability, supporting the structural findings. The non-ribosomal code of Pls-A was determined to be (1)Asp^213^-(2)Ala^214^-(3)Glu^217^-(4)Ser^256^-(5)Ile^277^-(6)Gly^279^-(7)Thr^301^-(8)Val^309^-(9)Val^310^-(10)Lys^495^, and the (3)Glu^217^ residue directly forms a salt bridge with the amino group of the lysine substrate’s side chain [34] (Figure 5E). This (3)Glu^217^ residue in the non-ribosomal code is also conserved in the A2 and A3 domains of PvdL(ORF3) in *P. fulva*, strongly suggesting lysine selectivity in these domains (Table 5). Further biochemical validation is required to confirm the substrate specificity. In particular, the stereochemistry of the incorporated amino acids (L- or D-form) in non-ribosomal peptide synthesis was inferred from the presence or absence of epimerization (E)-domains in each module of the PvdL. However, the stereochemical configuration cannot be determined using LC-MS/MS analysis alone, and this remains an important subject for future investigation.

## 4. Conclusions

This study characterized the biosynthetic mechanism and structural diversity of PVDs produced by *P. fulva*. Initially, antiSMASH was used for genomic bioinformatic analysis to predict the PVD structure, but the sequence of the three C-terminal residues could not be determined. The subsequent detailed structural analysis by LC-MS/MS revealed modified residues, such as AcOrn(OH) and cOrn(OH), which are capable of forming Fe^³+^-binding sites, as well as PVD isoforms with different chromophore side chains. Furthermore, the LC-MS-based comparison of the PVD molecular diversity among *Pseudomonas* species showed that the molecular mass patterns of these PVD isoforms are unique to *P. fulva*.

PVDs are known to play critical roles in iron acquisition competition among microorganisms. Although the underlying causes of their sequence diversity remain to be fully elucidated, horizontal gene transfer has been proposed as a contributing factor, based on patterns of codon usage and tetranucleotide composition [46]. In environments with limited iron availability, producing a siderophore structurally distinct from common types could confer a competitive advantage to bacteria.

Compared with the C-terminal region of the closely related *P. putida* PVDs, a marked divergence was identified in that of *P. fulva* PVDs. Since the N-terminal region is essential for receptor binding (e.g., FpvA [47]), the C-terminal region may be under relaxed selective pressure, allowing for species-specific diversification. In a manner similar to that of other PVDs, the presence of a Lys-selective A-domain in *P. fulva* may reflect genetic exchange events, such as horizontal gene transfer, although direct evidence is lacking [46].

Furthermore, the adjacent A2 and A3 domains in PvdL(ORF3), which introduce a lysine repeat sequence, share a high level of sequence identity (95%), suggesting that they may have arisen through domain duplication. Non-ribosomal peptide synthetases (NRPSs) are multidomain enzymes composed of modules, such as adenylation, peptidyl carrier, and condensation domains (Figure 1), and it has been reported that repeated domains within a single gene can often have different evolutionary origins [48]. This observation implies that domain duplication and the generation of paralogs within the genome have contributed significantly to the structural and functional diversification of NRPSs. Indeed, the evolution of NRPS domains is hypothesized to involve a gene duplication–deletion mechanism [48], and such domain duplication may also play a role in shaping PVD diversity in *P. fulva*. However, the specific functional and evolutionary contributions of these duplicated domains require further investigation.

This study has two main limitations. First, although the molecular structure of *P. fulva* PVDs was clarified, their physiological roles in iron acquisition remain to be tested. Second, while sequence diversity may be associated with horizontal gene transfer and gene duplication, the evolutionary mechanisms underlying these observations remain speculative and await experimental validation.

## Figures and Tables

**Table 1:** Summary of next-generation sequencing results for the *P. fulva* NBRC16639 genome.

Base	Count	Percentage
A	939,138	19.10%
T	1,499,495	30.50%
G	1,528,630	31.10%
C	950,693	19.30%
GC	3,028,125	61.60%
All	4,917,956	100.0%
Coding regions predicted by RAST		
Coding sequences (protein-coding regions)		4400
Misc RNA (other RNA-coding regions)		68
rRNA (ribosomal RNA-coding regions)		68
tmRNA (transfer-messenger RNA)		1
tRNA (transfer RNA-coding regions)		76

**Table 2:** Mass values for PVD isoforms A–D.

Peak	[M+H]^+^
A	1220.56
B	1205.54
B’	1204.57
C	1187.52
D	1131.50

**Table 3:** Amino acid analysis results for PVD isoform A.

Amino Acid	Amount (nmol)	Ratio
Asp	2.94	1.01
Ser	5.84	2
Orn	3.28	1.12
Lys	6.22	2.13

**Table 4:** LC-MS analysis results for culture supernatants from different *Pseudomonas* NBRC strains.

Strain	Observed Mass	Sequence	Ref.
*P. fulva* (NBRC:16637)	1115.47 1187.53 1204.56 1220.55 1238.56	Asp-Ser-Ser-AcOHOrn-Lys-Lys-cOHOrn	This study
*P. fulva* (NBRC:16639)	1131.50 1187.53 1204.56 1220.55	Asp-Ser-Ser-AcOHOrn-Lys-Lys-cOHOrn	This study
*P. parafulva* (NBRC:16635)	1190.48 1191.49 1206.50 1207.48	Asp-Orn-(OHAsp-Dab)-Gly-Ser-cOHOrn	[11]
*P. parafulva* (NBRC:16636)	1188.45 1206.47	Not assigned	
*P. putida* (NBRC:3778)	1368.51 1388.57 1404.57	Not assigned	[43]
*P. putida* (NBRC:12996)	1089.41 1091.42 1107.42 1122.43	Asp-εLys-OHAsp-Ser-Ala-Ser-cOHOrn	[44]
*P. putida* (NBRC:14164)	1319.50 1336.52 1337.51 1352.52	Ser-Gln-Dab-Asp-Thr-Gly-Asp-Thr-Thr-Gly	[11]
*P. putida* (NBRC:14671)	1342.58 1358.58 1359.58 1374.59	Not assigned	
*P. putida* (NBRC:14796)	1012.47 1150.42 1166.42 1184.43	Asp-Orn-Dab-Gly-Ser-Ser-OHAsp-Thr	[43]
*P. putida* (NBRC:15366)	1305.52 1321.51 1323.53 1338.54 1339.52 1352.55 1354.53 1368.55 1369.53	Asp-Lys-OHAsp-Ser-Ala-Thr-Thr-Thr-cOHOrn	[11]
*P. putida* (NBRC:100650)	1056.40 1073.42 1074.41 1092.42	Asp-Orn-(OHAsp-Dab)-Gly-Ser-cOHOrn	[11]
*P. putida* (NBRC:109349)	1187.49 1199.49 1217.50 1235.51 1251.52	Asp-εLys-OHAsp-Ser-Gly-aThr-Lys-cOHOrn	[45]
*P. putida* (NBRC:110474)	1323.53 1338.54 1339.52 1354.53 1356.55 1372.58	Ser-Gln-Dab-Asp-Thr-Gly-Asp-Thr-Thr-Gly	[11]

**Table 5:** Comparison of the non-ribosomal codes of the A-domains of 26 NRPSs that use lysine as a substrate.

Species	Domain	Signature
*Pseudomonas fulva*	PvdL(ORF3)A2	D A E D H G T V T K
*Pseudomonas fulva*	PvdL(ORF3)A3	D A E D H G T V T K
*Pseudomonas protegens Pf-5*	PvdD A3	D A E D N G T V S K
*Pseudomonas tolaasii*	APU91751 A6	D A E S V G T I I K
*Streptomyces albulus NBRC14147*	Pls-A	D A E S I G T V V K
*Xenorhabdus miraniensis*	Xmir_01407 A6	D A E S I G T V I K
*Planktothrix agardhii NIVA-CYA 126* */8*	apnA A2	D A E D I G S V V K
*Nostoc punctiforme PCC 73102*	Npun_F2460 A2	D A E D I G S V I K
*Stigmatella aurantiaca Sg a15*	mxcG A1	D A E D I G T V V K
*Anabaena sp. 90*	aptA1 A2	D T E D I G S V I K
*Anabaena sp. 90*	aptA2 A2	D T E D I G S V V K
*Mycobacterium tuberculosis H37Rv*	Rv0101 A1	D I E D V G S V V K
*Bacillus licheniformis*	bacB A1	D A E S I G S V C K
*Paenibacillus larvae subsp. larvae DSM 25430*	ERIC2_c18070 A1	D M E D V G S V D K
*Paenibacillus sp. OSY-SE*	pbtB A2	D V G D V G S I D K
*Streptomyces fradiae*	lptC A1	D A D D A G T V D K
*Streptomyces sp. NRRL F-4415*	gobR A2	D A D D G G F V D K
*Uncultured bacterium*	mlcL A1	D T D D M G Y V D K
*Streptomyces albus subsp. chlorinus*	FM076_21195 A1	D T E D M G Y V D K
*Streptomyces viridosporus ATCC 14672*	cip22 A5	D T D D M G F I D K
*Burkholderia sp. B8(2020)*	necA A1	D T E N I G T I S K
*Mycobacterium tuberculosis H37Rv*	MbtF A1	D A Q D A G C V E K
*Myxococcus virescens*	benD A1	D N E S G G T V A K
*Brevibacillus laterosporus*	BogB A2	D S G P S G A V D K
*Brevibacillus laterosporus*	BogC A4	D A G P S G A V D K
*Streptomyces filamentosus NRRL 15998*	SSGG_00679 A2	D V F E S G G V A K

## Data Availability

The original contributions presented in this study are included in the article. Further inquiries can be directed to the corresponding author.

## Abbreviations

The following abbreviations are used in this manuscript:

PVD	pyoverdine
A-domain	adenylation domain
NRPS	non-ribosomal peptide synthetase
C-domain	condensation domain
PCP	peptidyl carrier protein
Pvd	pyoverdine biosynthesis protein
Pls-A	ε-poly-L-lysine synthetase
ORF	open reading flame
HPLC	high-performance liquid chromatography
MS	mass spectrometry
T-domain	thiolation domain

## Appendix A

**Table A1:** MS/MS fragment ion measurement values and identification results for PVD isoform A.

Fragment Type	Chemical Formula	Observed *m*/*z* (Theoretical)
A_4_	C_26_H_32_N_7_O_12_	634.21 (634.210)
A_5_	C_33_H_44_N_9_O_15_	806.29 (806.295)
B_1_	C_17_H_17_N_4_O_6_	373.11 (373.114)
B_2_	C_21_H_22_N_5_O_9_	488.14 (488.141)
B_3_	C_24_H_27_N_6_O_11_	575.17 (575.173)
B_4_	C_27_H_32_N_7_O_13_	662.20 (662.205)
B_5_	C_34_H_44_N_9_O_16_	834.29 (834.290)
B_6_	C_40_H_56_N_11_O_17_	962.39 (962.385)
B_7_	C_46_H_68_N_13_O_18_	1090.48 (1090.480)
Y″_2_	C_11_H_23_N_4_O_3_	259.18 (259.176)
Y″_5_	C_27_H_52_N_9_O_9_	646.39 (646.388)
Y″_6_	C_30_H_57_N_10_O_11_	733.42 (733.420)
Y″_7_	C_34_H_62_N_11_O_14_	848.44 (848.447)

**Table A2:** MS/MS fragment ion measurement values and identification results for PVD isoform B.

Fragment Type	Chemical Formula	Observed *m*/*z* (Theoretical)
A_4_	C_26_H_32_N_7_O_11_	619.20 (619.215)
B_1_	C_17_H_17_N_4_O_5_	357.12 (357.119)
B_2_	C_21_H_22_N_5_O_8_	472.15 (472.146)
B_3_	C_24_H_27_N_6_O_10_	560.16 (559.178)
B_4_	C_27_H_32_N_7_O_12_	646.21 (646.210)
B_5_	C_34_H_44_N_9_O_15_	818.30 (818.295)
B_6_	C_40_H_56_N_11_O_16_	946.39 (946.390)
B_7_	C_46_H_68_N_13_O_17_	1075.47 (1074.485)
Y″_2_	C_11_H_23_N_4_O_3_	259.18 (259.176)
Y″_6_	C_30_H_57_N_10_O_11_	733.42 (733.420)
Y″_7_	C_34_H_62_N_11_O_14_	848.44 (848.447)

**Table A3:** MS/MS fragment ion measurement values and identification results for PVD isoform C.

Fragment Type	Chemical Formula	Observed *m*/*z* (Theoretical)
A_3_	C_23_H_25_N_5_O_9_	514.92 (514.165)
A_4_	C_26_H_30_N_6_O_11_	601.19 (601.197)
B_1_	C_17_H_15_N_3_O_5_	340.09 (340.101)
B_2_	C_21_H_20_N_4_O_8_	455.12 (455.128)
B_3_	C_24_H_25_N_5_O_10_	542.15 (542.160)
B_4_	C_27_H_30_N_6_O_12_	629.18 (629.192)
B_5_	C_34_H_42_N_8_O_15_	801.27 (801.276)
B_6_	C_40_H_54_N_10_O_16_	929.36 (929.371)
B_7_	C_46_H_66_N_12_O_17_	1057.46 (1057.466)
Y″_2_	C_11_H_23_N_4_O_3_	259.18 (259.176)
Y″_4_	C_24_H_47_N_8_O_7_	559.18 (559.356)
Y″_5_	C_27_H_52_N_9_O_9_	646.21 (646.388)
Y″_6_	C_30_H_57_N_10_O_11_	733.42 (733.420)
Y″_7_	C_34_H_62_N_11_O_14_	848.45 (848.447)

**Table A4:** MS/MS fragment ion measurement values and identification results for PVD isoform D.

Fragment Type	Chemical Formula	Observed *m*/*z* (Theoretical)
A_4_	C_23_H_26_N_6_O_10_	545.16 (545.170)
B_2_	C_18_H_16_N_4_O_7_	399.09 (399.101)
B_3_	C_21_H_21_N_5_O_9_	486.13 (486.133)
B_4_	C_24_H_26_N_6_O_11_	573.16 (573.165)
B_5_	C_31_H_38_N_8_O_14_	746.25 (746.250)
B_6_	C_37_H_50_N_10_O_15_	873.34 (873.345)
B_7_	C_43_H_62_N_12_O_16_	1001.43 (1001.440)
Y″_6_	C_30_H_57_N_10_O_11_	733.42 (733.420)
